# Nogo-A Expression in the Brain of Mice with Cerebral Malaria

**DOI:** 10.1371/journal.pone.0025728

**Published:** 2011-09-29

**Authors:** Peter Lackner, Ronny Beer, Gregor Broessner, Raimund Helbok, Karolin Dallago, Michael W. Hess, Kristian Pfaller, Christine Bandtlow, Erich Schmutzhard

**Affiliations:** 1 Department of Neurology, Innsbruck Medical University, Innsbruck, Austria; 2 Division of Histology and Embryology, Innsbruck Medical University, Innsbruck, Austria; 3 Division of Neurobiochemistry, Innsbruck Medical University, Innsbruck, Austria; Museum National d'Histoire Naturelle, France

## Abstract

Cerebral malaria (CM) is associated with a high rate of transient or persistent neurological sequelae. Nogo-A, a protein that is highly expressed in the endoplasmic reticulum (ER) of the mammalian central nervous system (CNS), is involved in neuronal regeneration and synaptic plasticity in the injured CNS. The current study investigates the role of Nogo-A in the course of experimental CM. C57BL/6J mice were infected with *Plasmodium berghei ANKA* blood stages. Brain homogenates of mice with different clinical severity levels of CM, infected animals without CM and control animals were analyzed for Nogo-A up-regulation by Western blotting and immunohistochemistry. Brain regions with Nogo-A upregulation were evaluated by transmission electron microscopy. Densitometric analysis of Western blots yielded a statistically significant upregulation of Nogo-A in mice showing moderate to severe CM. The number of neurons and oligodendrocytes positive for Nogo-A did not differ significantly between the studied groups. However, mice with severe CM showed a significantly higher number of cells with intense Nogo-A staining in the brain stem. In this region ultrastructural alterations of the ER were regularly observed. Nogo-A is upregulated during the early course of experimental CM. In the brain stem of severely affected animals increased Nogo-A expression and ultrastructural changes of the ER were observed. These data indicate a role of Nogo-A in neuronal stress response during experimental CM.

## Introduction

A major cause of morbidity and mortality of *Plasmodium falciparum* malaria is cerebral malaria (CM). It presents as a diffuse encephalopathy with alteration of consciousness, ranging from drowsiness to deep coma and is frequently accompanied by seizures [1]. The pathophysiological mechanisms of CM are yet not fully understood. Most researchers agree that the immune response of the host is a critical factor in the pathogenesis of CM — in particular in the murine model [2], [3]. In addition neuronal damage, as evidenced by chromatolysis in large nerve cells, swelling and vacuolation of the cytoplasm and loss of Nissl granules and glial degeneration, have been described [4], [5]. Neuronal damage is at least in part attributable to apoptotic mechanisms [6]. A deeper understanding of the upstream effectors would be necessary for the development of effective adjunctive treatment strategies aiming at the reduction of the high mortality of CM and the amelioration of neurological sequelae which are still observed in approximately 10% of the survivors [7]. In an even higher percentage of survivors neurocognitive sequelae are expected [8]. Recently, neurodegeneration as well as axonal damage have been identified as potential contributors and were shown to occur in the murine model as well as in human CM [9]–[11].

One CNS myelin protein which has gained publicity with respect to neuronal regeneration is Nogo-A. Nogo belongs to the reticulon family of proteins which are highly expressed in the endoplasmic reticulum (ER). Nogo-A is the largest splice variant of the Nogo gene, which gives rise to three major protein products, Nogo-A, -B, and -C, by both — alternative splicing and alternative promoter usage [12]. Nogo-A is exclusively expressed in the mammalian CNS by oligodendrocytes and certain neurons. Nogo-A was first identified as an antigen recognized by a monoclonal antibody against myelin extract [13] and later found to inhibit neurite outgrowth in the injured mammalian CNS by signaling involved with the extracellular presentation of Nogo-A and the binding to its receptor.

However, in the adult central nervous system, Nogo and other reticulons are also expressed within the neuronal endoplasmic reticulum (ER) [14]. In this regard Nogo-A might be co-regulated with ER-associated chaperones and heat-shock protein, serving as neuroprotectant by preconditioning neurons and glia against ER stress increasing their resistance to apoptotic insults [14]–[16]. Recent reports have indicated that Nogo-A protein level is up-regulated in several animal models for CNS injury, i.e. rat traumatic brain injury, rat hypoxia-ischemia and focal cerebral ischemia [17]–[19]. In addition Nogo-A has been shown to play an important role in experimental allergic autoencephalitis the mouse model of multiple sclerosis [20]. However to date Nogo-A expression has not been studied in infectious diseases of the CNS.

The current study aimed to evaluate the presence and potential alterations of Nogo-A expression in different clinical stages of experimental CM as a model disease for infectious and inflammatory pathology of the neurovascular compartment.

## Results

Thirty-three of forty-three infected animals developed signs of CM between day 4 and day 9 post infection with levels of parasitemia between 5% and 15% (CM group). Ten animals survived this period and were killed at day 11 post infection. Since these mice did not show marked neurological signs they served as an additional control group (NCM group). Parasitemia levels in the early course of the infection were highly similar between the two groups (data not shown).

### Western blot analysis

**Figure 1:**
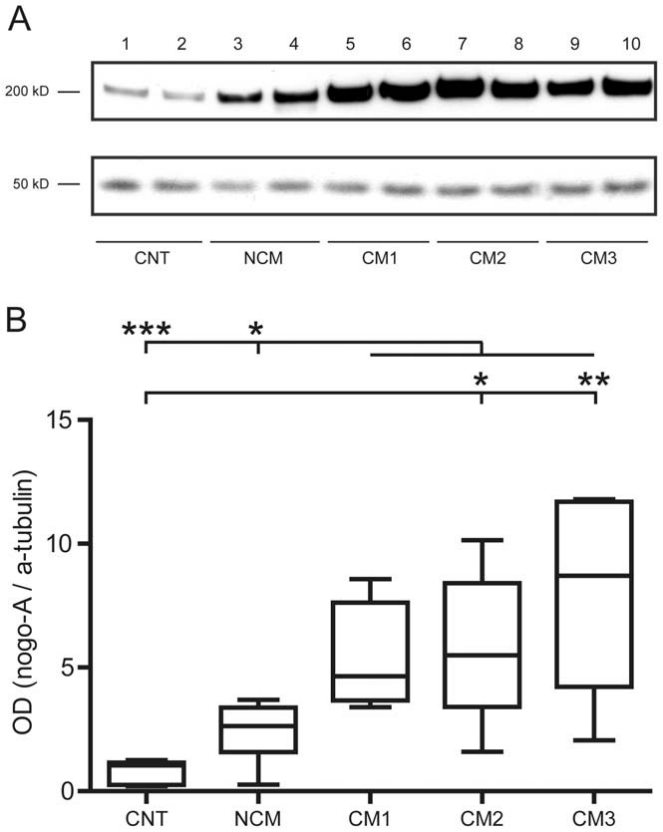
Representative Western blot (A) for Nogo-A (200 kDa) and alpha-tubulin (50 kDa) and densitometric analysis of western blot experiments (B) in mice with different stages of cerebral malaria (mild, CM1 n = 4; moderate, CM2 n = 9; severe CM3 n = 6), infected mice without neurological involvement (NCM n = 6) and uninfected control animals (CNT n = 5). A significant increase of densitometric measures for Nogo-A was observed in animals with CM compared to NCM and non-infected control animals. Comparing subgroups of animals with different clinical levels of severity CM2 and CM3 mice showed significantly higher Nogo-A levels compared to CNT mice. ***, p>0.001; **, p>0.01; *, p<0.05.

In order to determine whether Nogo-A expression is upregulated in the course of CM, brain extracts of mice with different levels of clinical severity were examined (total n = 30, CM1 n = 4, CM2 n = 9, CM3 n = 6, NCM n = 6, CNT n = 5). Mice with CM showed significantly higher densitometric measures for Nogo-A compared to NCM animals (p<0.05) and non-infected control animals (p<0.001). Infected mice without neurological signs (NCM) showed only mildly (not significantly) elevated levels of Nogo-A compared to CNT mice. Subsequent analyses of mice with different clinical stages of CM yielded a significant increase of densitometric measures for Nogo-A in moderately to severely affected animals (CM2, CM3) (Fig.1A–B). In mice with CM, correlation analysis did not reveal a significant association between the cumulative SHIRPA score and OD values for Nogo-A (data not shown).

### Immunohistochemistry

**Figure 2:**
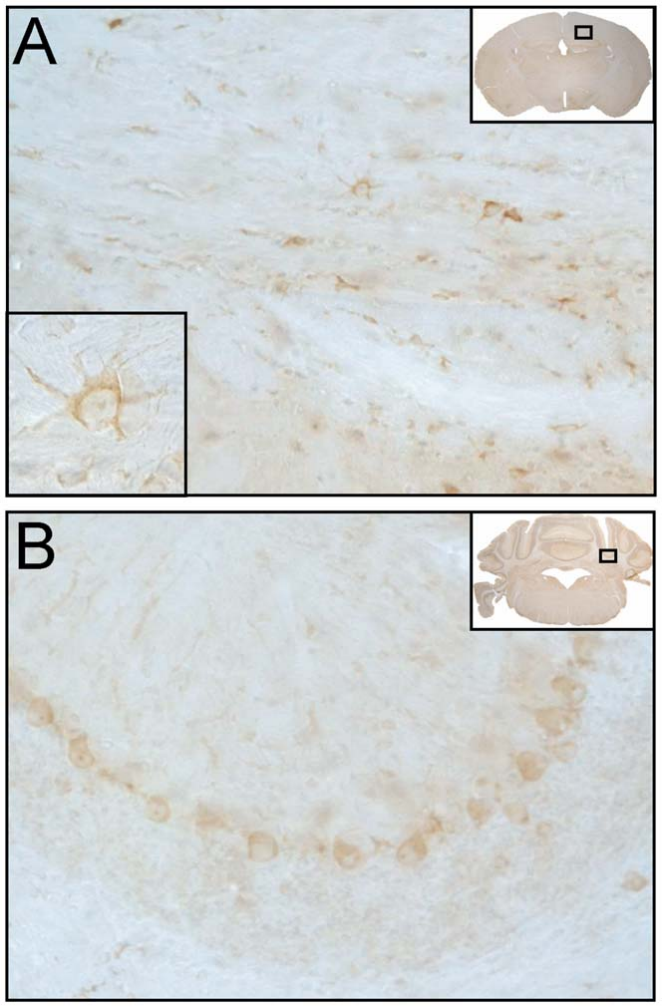
Representative micrographs of a non infected control animal. A: Corpus callosum (bregma -2); B: Cerebellum (bregma -6). Positive immunolabeling for Nogo-A was observed in cells showing morphological characteristics of oligodendrocytes (figure 2A) and neurons (figure 2B). Framed areas in the respective inserts delineate regions of interest presented in the micrographs. Magnifications: A: 20x, left inlay 100x; B: 20x.

**Figure 3:**
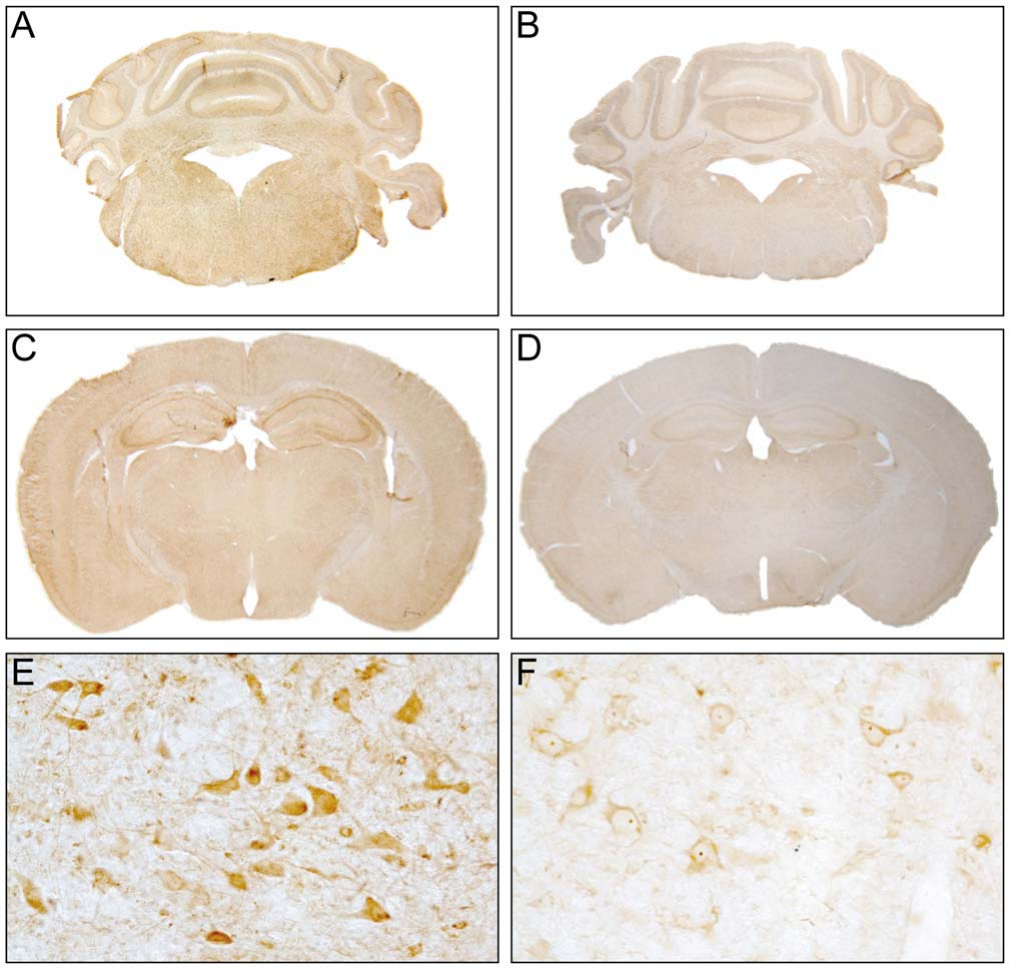
Representative micrographs of an animal with severe CM (A, C, E) and a non infected control animal (B, D, F). A, B, E, F: bregma -6 (cerebellum and brainstem); C, D: bregma -2 (cortex, hippocampus and thalamic nuclei). In CNT animals, brain sections showed mild labeling for Nogo-A (B, D, F). In CM animals more intense neuronal and oligodendroglial Nogo-A labeling was observed (A, C, E) especially in the brainstem (E). Magnifications: A–D: 1x, E-F: 20x.

**Figure 4:**
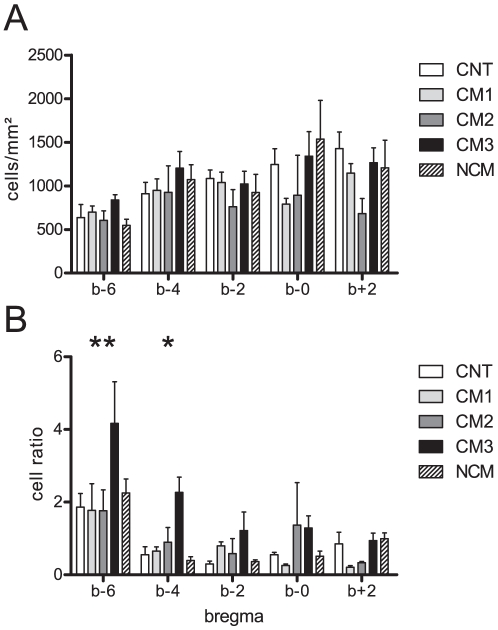
Stereological analysis of the number of Nogo-A positive cells per mm^2^ (A) and the ratio of Nogo-A positive cells per mm^2^ with intense labeling in relation to mildly labeled cells (B) in defined regions of the brain (bregma +2, 0, -2, -4, -6 mm). The total amount of parenchymal cells positive for Nogo-A did not differ significantly between the studied groups (A). In the brain stem animals with severe CM showed a significantly higher number of neurons and oligodendrocytes with intense Nogo-A labeling (B). **, p<0.001 for the comparison CM3 vs. CM2, CM1, CNT and p<0.01 for the comparison CM3 vs. NCM; *, p<0.01 for the comparison CM3 vs. NCM and p<0.05 for the comparison CM3 vs. CM1, CNT. Mean and SEM are shown.

Brains were examined rostrocaudally from bregma +2 to -6 mm. Cells labeled by antibodies against Nogo-A were observed in all analyzed samples and brain regions of CNT and CM animals. Nogo-A labeling was observed in cells showing morphological characteristics of oligodendrocytes (figure 2A) and neurons (figure 2B). In CNT animals, brain sections showed mild labeling for Nogo-A (figure 2, figure 3B, D, F). In CM animals more intense neuronal and oligodendroglial Nogo-A labeling was observed (figure 3A, C, E), especially in the brainstem. In order to quantify these differences morphometric analyses applying stereology were performed. The number of Nogo-A positive cells per mm^2^ with mild (figure 3F) or intense labeling respectively (figure 3E) were counted in defined regions of the brain (bregma +2, 0, -2, -4, -6 mm). The total amount of parenchymal cells positive for Nogo-A irrespective of labeling intensity did not differ significantly between the studied groups (figure 4A). In frontal parts of the brain higher densities of Nogo-A positive cells than in the brain stem or in cerebellum were observed (figure 4A). In the brain stem, animals with CM3 showed a significantly higher number of neurons and oligodendrocytes with intense Nogo-A labeling as compared to CNT, NCM, CM1 and CM2 animals. (figure 4B, figure 3A, C, E).

### Ultrastructural Analysis

**Figure 5:**
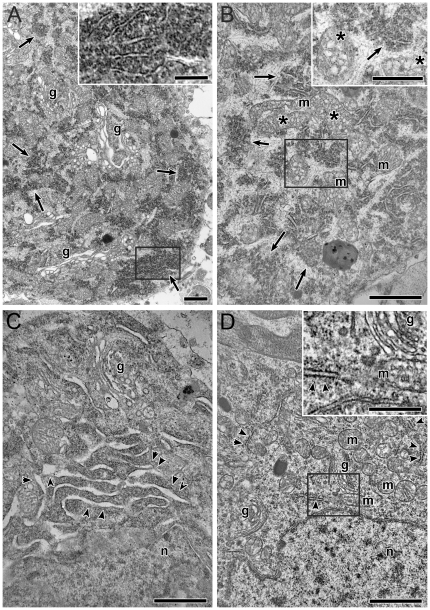
Transmission electron microscopic micrographs of animals with CM (A-C) and an uninfected control animal (D). In animals with CM, ultrastructural changes indicating alterations of the endoplasmic reticulum (ER) were observed. In most neurons ribosomes and / or polyribosomes appeared conspicuously clustered (A-B, arrows). Some neurons even showed dilated ER (C, arrow-heads) and Golgi apparatus (A,C: g). Mitochondria (m) frequently showed altered cristae (B: asterisks). Control specimens from the same brain regions did not show signs of neuronal or oligodendroglial degeneration (D). Golgi apparatus are marked by g; mitochondria by m, nuclei by n. Bars in A, B, C, D correspond to 1 µm. Framed areas in A, B, D are shown at higher magnification in the respective inserts, where the bars correspond to 0.5 µm.

To verify whether the increased neuronal Nogo-A expression in rostral and caudal brainstem in animals with CM is also reflected by morphological changes, representative samples from these regions were analyzed by transmission electron microscopy. In animals with CM, clearly recognizable alterations of the endoplasmic reticulum were regularly observed. In most neurons ribosomes and/or polyribosomes appeared conspicuously clustered (figure 5A–B). Some neurons even showed dilated ER and Golgi apparatus (figure 5A, C). Mitochondria frequently showed dilated cristae (figure 5B). Control specimens from the same brain regions did not show signs of neuronal or oligodendroglial degeneration (figure 5D).

## Discussion

Nogo-A is upregulated in the brain of mice with CM during the early phase of the neurological syndrome. While the total amount of brain cells with Nogo-A immunolabeling did not increase, the neuronal and oligodendroglial Nogo-A signal was markedly intensified especially in the brainstem of mice with CM. In this brain region ultrastructural alterations suggestive for ER stress were observed, which might indicate that Nogo-A is involved in the cellular stress response triggered of by CM.

The role of Nogo-A in the inhibition of axonal sprouting in the injured adult CNS has been studied extensively [21], [22]. In vitro, presentation of Nogo-A on the cell surface and binding to the neuronal Nogo receptor is required for the inhibitory effect [23]. Nogo-A has been shown to be upregulated in a variety of disease models for acute and chronic CNS injury and seems to be associated with impaired neuronal regeneration and synaptic plasticity [17]–[19], [ 24]. In cerebral malaria focal neurological signs and symptoms as well as neurocognitive deficits are observed in a high percentage of survivors [8]. Although neurological sequelae are reversible in some patients, others suffer from persisting disabilities. Especially children are most severely affected [25]. Inhibition of Nogo-A has been shown to increase regeneration after experimental spinal cord injury [26], and to ameliorate neurocognitive deficits after experimental traumatic injury [27]. In the current study Nogo-A upregulation was observed in the very early stage of the neurological syndrome. At this stage clinical alterations in murine CM are mainly confined to altered consciousness and alertness [28]. One might hypothesize that these clinical signs are at least in part attributable to disturbed synaptic plasticity due to Nogo-A upregulation and that inhibition of Nogo-A activity could lead to a better functional outcome. However, the data in traumatic brain injury concerning genetic deletion or pharmacological inhibition of Nogo-A and neurocognitive recovery are conflicting [29], [30]. Therefore also in CM a major role of Nogo-A as a repressor of axonal sprouting and plasticity seems unlikely. The increased expression of Nogo-A could rather relate to intracellular functions common to the reticulon protein family [31]. Like other members of the reticulon family, Nogo-A is associated with the ER and, similarly to RTN-1, it might be involved in protein transfer, packing and trafficking [32], [33]. It has been shown recently that Nogo-A is a novel regulator of the ER chaperone protein disulfide isomerase (PDI), and that through PDI, Nogo-A can protect mice against neurodegeneration that characterizes amyotrophic lateral sclerosis [16]. In the current study, the ultrastructure of neurons in the brainstem of CM animals was altered. Dilation of the ER indicated ER stress. In addition, mitochondria frequently showed altered cristae. Similar morphological patterns have been observed in ALS but also in an animal model of intoxication (Manganese) as well as after experimental ischemia [34]–[36]. These features, are therefore, considered as unspecific indicators for cellular stress in general and ER stress in particular. In addition, conspicuously clustered ribosomes / polyribosomes were observed in our CM samples. This could indicate that also protein transport is impaired during CM [36]. In this respect the upregulation of Nogo-A might be interpreted as a cellular counter-action to inflammatory stress which is known to occur in murine CM [37]. In line with our observations data from experimental autoimmune encephalitis and human multiple sclerosis suggest that Nogo-A has an important function in inflammatory brain processes [20], [38]. The exact role of Nogo-A in brain inflammation however remains to be resolved. In this respect further studies are required in other model diseases (e.g. bacterial meningitis).

In addition to inflammatory processes also ischemia as a consequence of microcirculatory dysfunction has been suggested to play a role in murine and human CM [39]–[41]. Hypoxia / ischemia has been shown to induce Nogo-A expression [18], [19]. Whereas in the first days after focal stroke the total number of Nogo-A positive neurons decreased, the intensity of neuronal Nogo-A labeling increased. Our results show that Nogo-A expression in neurons but not the total number of neurons positive for Nogo-A is increased in the acute phase of CM. These observations support the hypothesis that at least in the early phase after insult Nogo-A upregulation might be regarded as unspecific cellular response to ischemic stress.

Another common feature of murine CM are epileptic seizures. Nogo-A mRNA levels have been shown to be elevated in the adult rat hippocampal neurons after kainate-induced seizures [42]. Furthermore, Nogo-A is induced in hippocampal neurons of patients with temporal lobe epilepsy [43]. Seizure induced upregulation could explain the observed increase in Nogo-A expression in late stage CM. This is certainly not the case for the very early increase of Nogo-A expression during a CM phase in which seizures are usually not observed.

The presented data suggest regional differences in the intensity of Nogo-A upregulation during CM. The brainstem showed more intense labeling than other regions of interest. Recently, we demonstrated a similar spatial distribution of a marker for neuronal apoptosis (i.e. caspase-3 activation) [6]. Importantly, another experimental study investigated the temporal and spatial expression of the proto-oncogene product c-fos, an indicator of cellular stress, and identified the brainstem as an area of particular vulnerability in CM [44]. It has been shown previously that Nogo-A labeling is particularly strong in brain stem nuclei [45]. The higher constitutive expression of Nogo-A in brain stem neurons and the upregulation of Nogo-A in this region under stress supports the idea that Nogo-A exerts an important regulatory function in this vital brain area in general and in particular during murine CM.

In conclusion, the current study shows that Nogo-A is upregulated during the early course of experimental CM with increased expression in the brain stem of severely affected animals where ultrastructural changes suggestive for ER stress were observed. These data suggest a role of Nogo-A in ER associated neuronal stress response and point to a novel function of Nogo-A in this model disease for severe immune-mediated pathology of the neurovascular compartment.

## Materials and Methods

### Ethics statement

Animal studies conformed to the Austrian guidelines for the care and use of laboratory animals and were approved by the Austrian Federal Ministry of Science and Research (permit number GZ 66.011/53-BrGT/2004).

### Animals and Treatment

A total of 54 six to eight weeks old C57BL/6J mice (Charles River, Sulzfeld, Germany) were used for this study during 3 subsequent experimental infections. Forty-three animals were infected intraperitoneally with 1*10^6^ parasitized red blood cells of a homologue donor, which had been infected with frozen polyclonal stocks of *Plasmodium berghei ANKA*. Eleven animals were used as non infected control animals (CNT). The clinical severity of the disease was assessed by the SHIRPA-score primary screen on baseline, day 5 and before death [28]. The primary screen comprises a battery of 40 simple tests for evaluating neuromuscular, spinocerebellar, sensory, neuropsychiatric and autonomic functions in mice by observational assessment. The values of the respective tests were summed up and the cumulative SHIRPA-score was calculated as described previously [28]. Healthy mice show a value of about 30 while moribund CM animals show values of about 10.

Parasitemia was monitored daily by thin blood smear from tail blood. Between day 6 and day 9 post infection 33 of the infected mice developed signs of CM and were killed at different clinical stages of CM as measured by the cumulative SHIRPA score (CM1 >25, CM2 < = 25 and > = 15, CM3 <15) or for ethical reasons as soon as their body temperature dropped to 30°C, a valid marker for imminent death [28]. Mice which did not develop cerebral malaria (NCM n = 10) were killed on day 11 post infection to minimize suffering, as it is well established that animals which do not develop CM until day 10 will die from overwhelming parasitemia about 3 weeks after infection. All animals were given a lethal dose of 0.5 ml (25 mg/ml) thiopental (Biochemie, Kundl, Austria) intraperitoneally. Deeply anesthetized mice were transcardially perfused with ice-cold phosphate buffered saline (PBS) for two minutes followed by ice-cold fixative solution for 15 min with a pressure controlled syringe pump (Fresenius-Kabi, Germany).

### Western blot

Animals (total n = 30, CM1 n = 4, CM2 n = 9, CM3 n = 6, NCM n = 6, CNT n = 5) were perfused with PBS for 2 min. Brain samples were processed as described previously [6]. The microdissected tissue was homogenized in ice-cold buffer (pH 7.5) containing 50mM Tris-Cl, 5mM EDTA, 50mM NaCl, 5mM DTT, 0.1% Np-40, 50mM NaF, 1mM PMSF, 1mM Na3VO4 plus a protease inhibitor cocktail (Roche, Mannheim, Germany) and centrifuged at 18500g for 20 min at 4°C. Protein balanced samples were analyzed using standard techniques. The top parts of the blots were probed with a mouse monoclonal antibody directed against Nogo-A (11C7; 1∶2000 in blocking solution) overnight at 4°C. 11C7 is directed against amino acids 623–640 of rat Nogo-A. It is specific for Nogo-A and reveals a single band at 190–210 kD in Western blots of human and murine brain tissue, and does not cross-react with any other member of the reticulon family [12]. To control and correct for equal loading, the bottom part of each blot was probed for alpha-tubulin (Sigma, 1∶20000 in blocking solution) overnight at 4°C. Antibody binding was visualized using Enhanced chemoluminescence reagents (Lumiglo™; Cell Signaling).

### Immunohistochemistry

After perfusion with 4% paraformaldehyde in PBS (0.1M), brains (total n = 20, CM1 n = 4, CM2 n = 4, CM3 n = 4, NCM n = 4, CNT n = 4) were postfixed in the same fixative for 6 hours and cryoprotected with 30% sucrose in PBS. Frozen tissues were cut into 20 µm and 40 µm thick coronal sections on a freezing cryotome (Leica Microsystems, Nussloch, Germany). 20 µm thick sections were mounted on SupraFrostPlus slides (Microm International, Walldorf, Germany) and air dried. 40 µm thick sections were kept in assorter buffer and stored at 4°C. The directly mounted slides were stained with Hematoxylin-Eosin according to a standard protocol and used for the qualitative analysis of the neuropathology of CM. 40 µm free-floating sections (40 µm) were blocked for endogenous peroxidase activity (20% methanol containing 1% H2O2) for 20 min and non-specific binding in blocking solution (10% normal goat serum, 10% bovine serum albumin in Tris-buffered saline containing 0.1% Triton X-100) for 1 hour at room temperature. Rabbit polyclonal antibody directed against Nogo-A (Zymed, CA, USA) was diluted 1∶1000 in blocking solution and permitted to bind overnight at 4°C. Biotinylated goat anti-rabbit antibody (Vector Laboratories, Burlingame, CA, USA) was then applied at a dilution of 1∶1000 for 1 h at room temperature. Antibody binding was visualized using Vectastain ABC kit (Vector Laboratories) and diaminobenzidine as chromogen according to the manufacturer brochure. Sections without primary antibodies were equally processed to control for unspecific binding.

### Ultrastructural analysis

After perfusion with 3% glutaraldehyde in cacodylate buffer (0.1M), brains for ultrastructural analysis (total n = 4) , were postfixed in 1% unbuffered aqueous osmium tetroxide for 2 hours at 4°C and dehydrated in graded ethanol series followed by infiltration with graded series of Epon epoxy resin. Semi-thin sections stained with toluidine blue were screened for areas of interest. Subsequently, 100 nm sections were cut on an ultramicrotome, contrasted with uranyl acetate and lead citrate and viewed with a CM120 transmission electron microscope (Philips, Eindhoven, The Netherlands).

### Morphometric analysis

For morphometric analysis anatomical regions of interest (ROI) between +2 and –6 bregma (±350 µm) were determined as previously described [28]. Stereology was applied using a computer-assisted image analysis system: Nikon E-800 microscope with a motorized stage and Stereo Investigator Software (MicroBrightField, Magdeburg, Germany). The optical fractionator stereological method was used to count parenchymal cells positive for Nogo-A [46]. One section per ROI from each animal was examined in a blinded way with objective ×100 with a counting frame of 70×50 µm and a sampling grid area of 700×1000 µm. Only extravascular cells with a clear labeling and morphological characteristics of neurons or glia were counted.

### Statistical analysis

Spearman rank correlation was calculated between OD values for Nogo-A and the cumulative SHIRPA score. OD values of Nogo-A were compared between groups by Kruskal-Wallis test and Dunn's multiple comparison test. Nogo-A positive brain parenchymal cells were compared between groups by two-way ANOVA with anatomical ROI and clinical severity group as factors. P-values were Bonferroni-corrected for multiple comparisons. All statistical analyses were done using GraphPad Prism version 5.00 (GraphPad Software, San Diego California USA).
